# Bidirectional relationship between metabolic dysfunction-associated steatotic liver disease and type 2 diabetes mellitus

**DOI:** 10.17179/excli2026-9462

**Published:** 2026-06-12

**Authors:** Alessandro Mantovani, Riccardo Morandin, Nicoletta Rolli, Elisa Molinaroli, Giovanni Targher

**Affiliations:** 1 Department of Medicine, University of Verona, Verona, Italy; 2 Metabolic Diseases Research Unit, IRCCS Sacro Cuore – Don Calabria Hospital, Negrar di Valpolicella, Italy

**Keywords:** MASLD, type 2 diabetes, liver fat, review

## Abstract

Metabolic dysfunction-associated steatotic liver disease (MASLD) and type 2 diabetes mellitus (T2DM) are two common, interconnected conditions that pose a major global health challenge. The worldwide prevalence of MASLD among individuals with T2DM exceeds 60 %, with a substantial proportion of cases having metabolic dysfunction-associated steatohepatitis (MASH) and an increased risk of liver-related complications, such as cirrhosis, liver failure, or hepatocellular carcinoma. The coexistence of MASLD and T2DM is also associated with poorer glycemic control and a higher risk of cardiovascular events, chronic kidney disease, and mortality. Notably, MASLD increases the risk of developing T2DM, with risk rising stepwise with liver disease severity, especially liver fibrosis. The close bidirectional relationship between MASLD and T2DM creates a vicious cycle that drives liver disease progression, worsens insulin resistance, and impairs glucose metabolism. This likely reflects shared underlying mechanisms, including insulin resistance, low-grade inflammation, lipotoxicity, adipose tissue dysfunction, and an altered gut-liver axis. Screening strategies are crucial for MASLD and T2DM, with current guidelines recommending assessment of liver fibrosis in all individuals with T2DM and regular screening for dysglycemia in those with MASLD. Pharmacological treatments, especially incretin-based therapies, sodium-glucose cotransporter 2 inhibitors, and resmetirom, show significant benefits across metabolic, hepatic, and extrahepatic outcomes. Overall, recognizing and addressing the bidirectional relationship between MASLD and T2DM is essential for better risk stratification, earlier intervention, and reduced long-term hepatic and extrahepatic complications. This narrative review summarizes current evidence on the bidirectional relationship between MASLD and T2DM, discussing epidemiological data, pathophysiological mechanisms, clinical implications, and therapeutic options.

See also the graphical abstract(Fig. 1).

## Introduction

Metabolic dysfunction-associated steatotic liver disease (MASLD) has become the most common chronic liver disease worldwide, posing a significant global public health challenge (Targher et al., 2025[79]; Tilg et al., 2026[82]). Epidemiological data indicate that MASLD affects up to one-third of adults worldwide (Feng et al., 2025[20]), and its prevalence is steadily rising alongside the global epidemics of obesity and type 2 diabetes mellitus (T2DM) (Targher et al., 2025[79]; Tilg et al., 2026[82]).

MASLD is diagnosed when hepatic steatosis occurs in combination with cardiometabolic risk factors, including overweight/obesity, elevated blood pressure, atherogenic dyslipidemia, prediabetes, or T2DM (Targher et al., 2021[75], 2025[79]; Tilg et al., 2026[82]). Among these, T2DM is the most clinically relevant metabolic comorbidity (Targher et al., 2021[75], 2025[79]; Tilg et al., 2026[82]). Epidemiological data consistently show that more than 65 % of individuals with T2DM have MASLD (Younossi et al., 2024[87]), underscoring the strong metabolic link between these conditions (Targher et al., 2021[75], 2025[79]; Tilg et al., 2026[82]). Individuals with T2DM also progress more rapidly to advanced forms of MASLD, including metabolic dysfunction-associated steatohepatitis (MASH), advanced fibrosis, cirrhosis, and hepatocellular carcinoma, than those without diabetes (Targher et al., 2021[75]; Vilar-Gomez et al., 2026[83]).

However, growing evidence indicates that the relationship between MASLD and T2DM is complex and bidirectional. Instead, MASLD itself increases the risk of new-onset T2DM (Mantovani et al., 2021[46]), likely through mechanisms involving insulin resistance, low-grade inflammation, lipotoxicity, and altered lipid metabolism (Targher et al., 2021[75]). Notably, among individuals with MASLD, the risk of incident T2DM increases progressively with the severity of liver fibrosis (Mantovani et al., 2021[46]).

Beyond liver-related complications, the coexistence of MASLD and T2DM is strongly associated with an increased risk of important extra-hepatic outcomes (Wild et al., 2017[85]; Younossi et al., 2024[87]; Han et al., 2025[28]), including fatal and nonfatal cardiovascular events (Mantovani et al., 2021[40]), chronic kidney disease (Mantovani et al., 2022[44]), and certain extra-hepatic cancers (Mantovani et al., 2022[45]). Therefore, MASLD is increasingly recognized as a multisystem metabolic disease rather than a condition limited solely to the liver (Byrne and Targher, 2015[7]; Targher et al., 2021[78]). In line with this, incretin-based therapies, including GLP-1 receptor agonists and newer multi-receptor agonists, are becoming an important and promising therapeutic option for MASLD/MASH, especially in individuals with T2DM or obesity (Targher et al., 2025[77]).

In this narrative review, we summarize and discuss current evidence on the bidirectional relationship between MASLD and T2DM, focusing on epidemiological data, pathophysiological mechanisms, clinical implications, and pharmacologic treatments.

## T2DM Increases Risk of MASLD Development and Progression

In a recent meta-analysis of 123 observational cohort studies involving about 2.22 million individuals with T2DM, Younossi et al. reported a global pooled prevalence of MASLD (detected by liver imaging or biopsy) of 65.3 % (95 % confidence interval 62.3 %-68.2 %) (Younossi et al., 2024[87]). Among patients with T2DM, the highest MASLD prevalence was observed in Eastern Europe (80.6 %), followed by the Middle East (71.2 %), with the lowest in Africa (53 %) (Younossi et al., 2024[87]). Notably, among patients with T2DM and liver biopsy data (n = 12 studies involving 2,733 patients), the global pooled prevalence of MASH, significant fibrosis, and advanced fibrosis was 66.4 %, 40.8 %, and 15.5 %, respectively (Younossi et al., 2024[87]). In people with T2DM, male sex, higher BMI, larger waist circumference, and increased plasma triglyceride levels are the main risk factors for hepatic steatosis (MASLD) (Mantovani et al., 2026[48]). Conversely, older age, higher BMI, and increased serum transaminase levels are the strongest predictors of MASLD-related liver fibrosis (Mantovani et al., 2026[48]).

Strong evidence also shows that individuals with T2DM are at higher risk of liver-related complications, including “at-risk MASH” (a term used to describe patients with MASH and significant fibrosis of stage 2 or higher) (Lazarus et al., 2025[35]), cirrhosis, hepatic decompensation, and HCC (Vilar-Gomez et al., 2026[83]; Simon et al., 2023[73]; Mittal et al., 2024[52]; Huang et al., 2023[31][32]; Castera et al., 2023[9]). In a prospective study of 530 individuals with T2DM, Mittal et al. found that the prevalence of “at-risk MASH” and cirrhosis (assessed by magnetic resonance imaging) was 13.6 % and 6.8 %, respectively (Mittal et al., 2024[52]). An observational study of 447 patients with MASLD and paired liver biopsies found that hepatic fibrosis progressed more rapidly in those with T2DM than in those without diabetes (Huang et al., 2023[32]). In an individual participant-level data meta-analysis of 2,016 adults with MASLD (736 with T2DM and 1,280 without T2DM), Huang et al. found that T2DM was associated with a significantly higher risk of hepatic decompensation (hazard ratio 3.29, 95 % confidence interval 2.21-4.90) and new-onset HCC (hazard ratio 7.72, 95 % confidence interval 2.61-22.87) over a median follow-up of 2.8 years, even after adjusting for risk factors and other potential confounders, including baseline liver stiffness measured by magnetic resonance elastography (Huang et al., 2023[31]).

The impact of T2DM on the risk of new-onset HCC is even more pronounced in individuals carrying the rs738409 *PNPLA3* (patatin-like phospholipase domain-containing protein 3) polymorphism, the genetic variant most strongly associated with MASLD and its more advanced forms (Sookoian et al., 2024[74]). In a case-control study of 257 patients with HCC and 494 controls, Hassan et al. found that the risk of HCC was significantly higher in individuals with T2DM who carried the *PNPLA3* rs738409 variant than in nondiabetic controls with the same variant (Hassan et al., 2013[30]). In a study of 671 individuals with MASH (444 adults and 227 children) who had at least 2 serial liver biopsies and were enrolled in different NASH-CRN studies and randomized controlled trials, Vilar-Gomez et al. found that three genetic factors (*PNPLA3* rs738409, *TM6SF2* rs58542926, and *HSD17B13* rs72613567), T2DM, and changes in BMI were strongly associated with histological progression of MASLD, and that T2DM modified the impact of genetic factors on histological liver outcomes (Vilar-Gomez et al., 2026[83]).

The coexistence of MASLD and T2DM further increases the risk of CVD events and all-cause mortality. In a cohort of nearly 650,000 South Korean middle-aged individuals with T2DM followed for a median of 6.2 years, those with persistent MASLD had a higher risk of heart failure (hazard ratio 1.28, 95 % confidence interval 1.25-1.32), myocardial infarction (hazard ratio 1.15, 95 % confidence interval 1.10-1.20), ischemic stroke (hazard ratio 1.14, 95 % confidence interval 1.09-1.19), and all-cause mortality (hazard ratio 1.11, 95 % confidence interval 1.09-1.14) than those who never had MASLD (Han et al., 2025[28]). Similarly, both incident and regressed MASLD were associated with increased risk of heart failure, myocardial infarction, stroke, and all-cause mortality (Han et al., 2025[28]). In another prospective cohort study of 134,368 individuals with T2DM, including 1,452 with MASLD and 1,707 with alcoholic liver disease, who were followed for a median of 4.3 years, MASLD (identified using ICD-9 and ICD-10 codes) was significantly associated with a higher risk of CVD events (hazard ratio 1.70, 95 % confidence interval 1.52-1.90) and all-cause mortality (hazard ratio 1.60, 95 % confidence interval 1.40-1.83) (Wild et al., 2017[85]).

Evidence also suggests that coexisting MASLD in individuals with T2DM is associated with poorer long-term glycemic control and increased hepatic and peripheral insulin resistance. In a small sample of 61 postmenopausal women with T2DM and MASLD, with baseline liver ultrasonography and vibration-controlled transient elastography (VCTE) in 2017 and follow-up data in 2022, Mantovani et al. found that the presence of MASLD and clinically significant fibrosis was associated with about a 4.5-fold higher risk of worse glycemic control at follow-up (defined as an HbA1c increase ≥ 0.5 % from baseline), even after adjustment for age, BMI, baseline HbA1c (or HOMA-estimated insulin resistance), and the use of glucose-lowering medications, such as pioglitazone, glucagon-like peptide-1 (GLP-1) receptor agonists, or sodium-glucose cotransporter 2 (SGLT2) inhibitors (Mantovani et al., 2022[49]). In another study of 230 patients with T2DM recruited from an endocrine clinic or primary care who underwent routine hepatology assessment (using VCTE with LSM and controlled attenuation parameter [CAP]), Patel et al. found that higher CAP values (hepatic steatosis) were associated with hemoglobin A1c ≥ 7 % and insulin treatment (Patel et al., 2018[58]). Conversely, among patients with T2DM, poor glycemic control has been associated with a higher risk of liver stiffness progression than good glycemic control (Zhou et al., 2026[91]). In addition, hepatic steatosis appears to be a key factor explaining interindividual variation in the daily insulin doses required to achieve good glycemic control in individuals with insulin-treated T2DM (Ryysy et al., 2000[65]).

Collectively, these findings underscore the importance of clinicians recognizing MASLD in individuals with T2DM. Once MASLD is diagnosed, clinicians should carefully assess for advanced disease, particularly the severity of liver fibrosis.

## MASLD Increases Risk of New-Onset T2DM

In a meta-analysis of 33 observational cohort studies involving 501,022 middle-aged individuals (30.8 % with MASLD) and 27,953 new cases of T2DM, Mantovani et al. reported that individuals with MASLD had approximately a 2.2-fold higher risk of incident T2DM than those without MASLD over a median follow-up of 5 years (random-effects hazard ratio 2.19, 95 % confidence interval 1.93-2.48). This increased risk of T2DM was independent of age, sex, adiposity measures, and other common metabolic risk factors. Notably, individuals with advanced MASLD, especially those with greater liver fibrosis, were also more likely to develop T2DM (random-effects hazard ratio 3.42; 95 % confidence interval 2.29-5.11) (Mantovani et al., 2021[46]). In this context, a retrospective cohort study of 396 Swedish patients diagnosed with MASLD by liver biopsy between 1971 and 2009, who had no T2DM at baseline, found that 51 % of those with histologic fibrosis stages F3-4 (advanced fibrosis) developed incident T2DM, compared with 31 % of those with fibrosis stages F0-2, over a mean follow-up of 18 years (Björkström et al., 2017[4]). Published data show that adding MASLD to traditional metabolic risk factors improves T2DM risk prediction in both sexes, with a greater benefit in women (Kim et al., 2022[34]). Mendelian randomization studies (using risk alleles in *PNPLA3* and other MASLD-related genetic variants) also indicate that genetically driven MASLD causally increases the risk of new-onset T2DM (Liu et al., 2022[37]; Ni et al., 2023[54]; Yu et al., 2023[89]). MASLD also predicts progression to T2DM in individuals with prediabetes at baseline (Busquets-Cortés et al., 2021[5]).

Some observational studies have also examined the risk of incident T2DM in relation to changes in MASLD status over time. In a cohort of 4,604 Japanese participants with two health check-ups at least 10 years apart, Yamazaki et al. found that improvement in MASLD (as measured by ultrasonography) was significantly associated with a lower incidence of T2DM (odds ratio 0.27, 95 % confidence interval 0.12-0.61), even after adjusting for age, sex, family history of T2DM, BMI, impaired fasting glucose, dyslipidemia, hypertension, and physical activity (Yamazaki et al., 2015[86]). Other observational cohort studies in Asian populations reported similar findings (Cho et al., 2019[14]; Chen et al., 2023[11]).

Overall, these findings indicate that individuals with MASLD have a substantially increased risk of developing T2DM, and this risk rises with more advanced liver disease, particularly higher stages of liver fibrosis. Therefore, these findings underscore the clinical importance of routine metabolic screening and risk assessment for all individuals with MASLD, especially those with advanced liver fibrosis, to enable earlier detection and targeted preventive interventions.

## Putative Pathophysiological Mechanisms Underlying the Bidirectional Relationship Between MASLD and T2DM

The close bidirectional relationship between MASLD and T2DM reflects a complex interplay of metabolic, proinflammatory and molecular mechanisms, many of which remain poorly understood (Anstee et al., 2013[3]; Tilg et al., 2017[81]; Ferguson and Finck, 2021[22]; Targher et al., 2021[75]; Wang et al., 2025[84]). MASLD and T2DM share pathophysiological pathways, and each can precede and worsen the other, creating a “vicious cycle” that accelerates disease progression and increases the risk of long-term hepatic and extrahepatic complications (Anstee et al., 2013[3]; Tilg et al., 2017[81]; Ferguson and Finck, 2021[22]; Targher et al., 2021[75]; Wang et al., 2025[84]).

A key mechanism underlying the bidirectional relationship between MASLD and T2DM is insulin resistance, which is central to both conditions (Anstee et al., 2013[3]; Tilg et al., 2017[81]; Ferguson and Finck, 2021[22]; Targher et al., 2021[75]; Wang et al., 2025[84]). In MASLD, hepatic insulin resistance increases gluconeogenesis and impairs glycogen synthesis, thereby increasing hepatic glucose production (Samuel et al., 2004[66]; Anstee et al., 2013[3]; Tilg et al., 2017[81]; Ferguson and Finck, 2021[22]; Targher et al., 2021[75]; Nogueira and Cusi, 2024[55]; Scoditti et al., 2024[71]; Wang et al., 2025[84]). At the same time, insulin resistance in adipose tissue increases lipolysis, raising the flux of free fatty acids (FFA) to the liver (Anstee et al., 2013[3]; Tilg et al., 2017[81]; Ferguson and Finck, 2021[22]; Targher et al., 2021[75]; Wang et al., 2025[84]). This excess FFA influx drives hepatic fat accumulation (Roden et al., 2000[64]), a hallmark of MASLD (Anstee et al., 2013[3]; Tilg et al., 2017[81]; Ferguson and Finck, 2021[22]; Targher et al., 2021[75]; Wang et al., 2025[84]). Consequently, hepatic fat accumulation exacerbates hepatic insulin resistance through lipotoxicity, contributing to systemic glucose dysregulation and increasing the risk of new-onset T2DM (Anstee et al., 2013[3]; Tilg et al., 2017[81]; Ferguson and Finck, 2021[22]; Targher et al., 2021[75]; Wang et al., 2025[84]).

Lipotoxicity is another important mechanism linking MASLD and T2DM (Anstee et al., 2013[3]; Tilg et al., 2017[81]; Ferguson and Finck, 2021[22]; Targher et al., 2021[75]; Wang et al., 2025[84]). The accumulation of toxic lipid intermediates, such as diacylglycerols and ceramides, may disrupt insulin signaling pathways (Petersen and Shulman, 2018[59]; Wang et al., 2025[84]). These lipid species activate protein kinase C isoforms, which impair insulin receptor signaling by inhibiting insulin receptor substrate activity (Anstee et al., 2013[3]; Tilg et al., 2017[81]; Ferguson and Finck, 2021[22]; Targher et al., 2021[75]; Wang et al., 2025[84]). This results in decreased glucose uptake in skeletal muscle and other peripheral tissues and increased hepatic glucose production (Anstee et al., 2013[3]; Tilg et al., 2017[81]; Ferguson and Finck, 2021[22]; Targher et al., 2021[75]; Wang et al., 2025[84]). Additionally, lipotoxicity induces mitochondrial dysfunction and oxidative stress, further damaging hepatocytes and pancreatic β-cells and worsening both liver disease and T2DM (Anstee et al., 2013[3]; Tilg et al., 2017[81]; Ferguson and Finck, 2021[22]; Targher et al., 2021[75]; Wang et al., 2025[84]).

Low-grade inflammation also plays a key role in the bidirectional relationship between MASLD and T2DM (Anstee et al., 2013[3]; Tilg et al., 2017[81]; Ferguson and Finck, 2021[22]; Targher et al., 2021[75]; Wang et al., 2025[84]). In MASLD, hepatic fat accumulation triggers a proinflammatory response characterized by Kupffer cell activation and increased release of multiple proinflammatory cytokines, including tumor necrosis factor-alpha, interleukin-6, and C-reactive protein (Anstee et al., 2013[3]; Tilg et al., 2017[81]; Ferguson and Finck, 2021[22]; Targher et al., 2021[75]; Wang et al., 2025[84]). These hepatic mediators contribute to systemic insulin resistance and impair pancreatic β-cell function (Accili et al., 2025[1]). Conversely, in T2DM, persistent hyperglycemia and elevated advanced glycation end-products (AGEs) further stimulate proinflammatory pathways (Khalid et al., 2022[33]; Accili et al., 2025[1]), exacerbating liver inflammation and promoting progression of MASLD to MASH, advanced fibrosis, and cirrhosis (Anstee et al., 2013[3]; Tilg et al., 2017[81]; Ferguson and Finck, 2021[22]; Targher et al., 2021[75]; Wang et al., 2025[84]).

Adipose tissue dysfunction is another key contributor to the bidirectional relationship between MASLD and T2DM. In people with obesity or metabolic syndrome, expanded visceral adipose tissue becomes dysfunctional, characterized by adipocyte hypertrophy, chronic hypoxia, and increased immune cell infiltration (Anstee et al., 2013[3]; Tilg et al., 2017[81]; Ferguson and Finck, 2021[22]; Targher et al., 2021[75]; Wang et al., 2025[84]). This dysfunction alters adipokine secretion, lowering plasma adiponectin (which has insulin-sensitizing and anti-inflammatory effects) and raising plasma leptin and resistin (which promote low-grade inflammation and insulin resistance) (Zhao et al., 2025[90]). The resulting imbalance in adipokine secretion further contributes to hepatic steatosis and systemic metabolic dysregulation (Anstee et al., 2013[3]; Tilg et al., 2017[81]; Ferguson and Finck, 2021[22]; Targher et al., 2021[75]; Wang et al., 2025[84]), linking MASLD and T2DM.

The gut-liver axis has emerged as an additional mechanism linking MASLD and T2DM. Alterations in gut microbiota composition (dysbiosis) increase intestinal permeability, allowing bacterial endotoxins such as lipopolysaccharide (LPS) to enter the portal circulation (Targher et al., 2021[75]; Tilg et al., 2021[80]). This promotes liver inflammation by activating toll-like receptors and contributes to insulin resistance (Targher et al., 2021[75]; Tilg et al., 2021[80]). In T2DM, intestinal dysbiosis may also affect glucose metabolism by modulating incretin hormone signaling and bile acid metabolism, further strengthening the link between MASLD and T2DM (Targher et al., 2021[75]; Yu et al., 2025[88]). GLP-1 plays a crucial role in linking MASLD and T2DM. Secreted by intestinal L-cells in response to nutrient intake, GLP-1 enhances glucose-dependent insulin secretion, inhibits glucagon release, and delays gastric emptying, thereby improving long-term glycemic control (Gribble and Reimann, 2021[27]; Drucker, 2025[18]). Additionally, GLP-1 improves hepatic metabolism by reducing hepatic de novo lipogenesis, promoting weight loss, and enhancing systemic insulin sensitivity (Gribble and Reimann, 2021[27]; Drucker, 2025[18]). An impaired incretin effect in individuals with T2DM may contribute to the progression of MASLD (Targher et al., 2021[75]), whereas pharmacological activation of the GLP-1 pathway reduces hepatic fat and inflammation (Targher et al., 2025[77]; Drucker, 2025[18]). Bile acids may also play a regulatory role in the bidirectional relationship between MASLD and T2DM (Targher et al., 2021[75]; Fuchs and Trauner, 2022[26]; Cadena Sandoval and Haeusler, 2025[8]). Beyond their primary role in lipid digestion, bile acids may act as signaling molecules through the Farnesoid X receptor (FXR) and the Takeda G protein-coupled receptor 5 (TGR5), thereby modulating glucose metabolism, insulin sensitivity, and hepatic lipid homeostasis (Targher et al., 2021[75]; Fuchs and Trauner, 2022[26]; Cadena Sandoval and Haeusler, 2025[8]). Dysregulation of bile acid signaling might therefore contribute to the development of MASLD and poor glycemic control (Targher et al., 2021[75]; Fuchs and Trauner, 2022[26]; Cadena Sandoval and Haeusler, 2025[8]).

Genetic and epigenetic factors may also contribute to the bidirectional relationship between MASLD and T2DM. Variants in genes such as *PNPLA3*, *TM6SF2 *(transmembrane-6 superfamily member 2), and *MBOAT7* (membrane-bound O-acyl transferase 7) have been associated with increased susceptibility to MASLD/MASH and may also affect glucose metabolism (Sookoian et al., 2024[74]; Vilar-Gomez et al., 2026[83]). Epigenetic changes, including DNA methylation and histone modifications, may be influenced by environmental factors, such as diet and physical activity, further modulating disease risk and progression (Sookoian et al., 2024[74]).

Finally, pancreatic β-cell dysfunction is another key link between MASLD and T2DM (Roden and Shulman, 2019[63]). In MASLD, increased hepatic glucose production and systemic insulin resistance place greater demands on pancreatic β-cells to secrete insulin (Anstee et al., 2013[3]; Tilg et al., 2017[81]; Ferguson and Finck, 2021[22]; Targher et al., 2021[75]; Wang et al., 2025[84]). Over time, this compensatory response fails, leading to β-cell exhaustion and the development of T2DM (Roden and Shulman, 2019[63]). Conversely, in established T2DM, persistent hyperglycemia and glucotoxicity may promote hepatic lipid accumulation and inflammation, thereby worsening MASLD (Anstee et al., 2013[3]; Tilg et al., 2017[81]; Ferguson and Finck, 2021[22]; Targher et al., 2021[75]; Wang et al., 2025[84]).

## Screening for MASLD in Patients With T2DM

**Figure 1:**
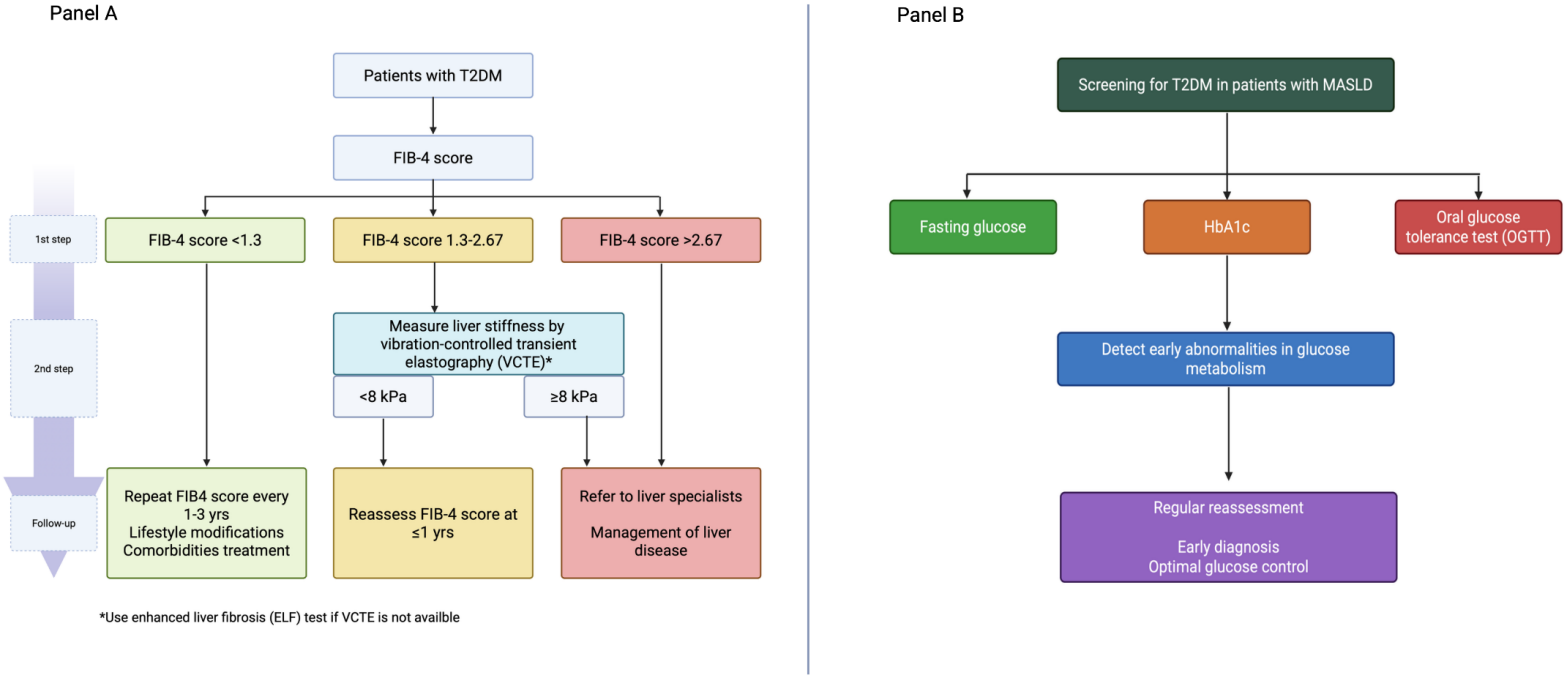
Graphical abstract: Diagnostic algorithm for detecting advanced liver fibrosis in individuals with type 2 diabetes mellitus (T2DM) (A), and screening strategies for diagnosing T2DM in individuals with MASLD (B), according to current guidelines. Created with https://BioRender.com

In individuals with T2DM, the primary goal of MASLD screening is to identify those with advanced liver fibrosis to prevent progression to cirrhosis, HCC, liver transplantation, and increased all-cause and liver-related mortality. Current guidelines from European and American hepatology societies and the American Diabetes Association (ADA) strongly recommend a two-step approach to screening for advanced liver fibrosis in individuals with T2DM (Figure 1(Fig. 1), Panel A) (Rinella et al., 2023[62]; EASL et al., 2024[19]; American Diabetes Association Professional Practice Committee for Diabetes*, 2025[2]; Cusi et al., 2025[17]). The first step is to calculate the FIB-4 index, typically performed by primary care clinicians or diabetes specialists (Rinella et al., 2023[62]; EASL et al., 2024[19]; American Diabetes Association Professional Practice Committee for Diabetes*, 2025[2]; Cusi et al., 2025[17]). The FIB-4 index is a validated, noninvasive score with high negative predictive value for ruling out advanced liver fibrosis. It is based on age, serum aspartate aminotransferase (AST), serum alanine aminotransferase (ALT), and platelet count (Byrne et al., 2018[6]; Feng et al., 2025[21]). Patients with a FIB-4 score < 1.3 (or < 2.0 if aged 65 or older) are considered at low risk for advanced liver fibrosis and can be managed in primary care or diabetes outpatient services, with periodic reassessment (Rinella et al., 2023[62]; EASL et al., 2024[19]; American Diabetes Association Professional Practice Committee for Diabetes*, 2025[2]; Cusi et al., 2025[17]). Patients with FIB-4 scores > 1.3 (or ≥ 2.0 in those aged ≥ 65 years) require further risk stratification with second-line noninvasive tests, such as vibration-controlled transient elastography (VCTE) or the enhanced liver fibrosis (ELF) test (Rinella et al., 2023[62]; EASL et al., 2024[19]; American Diabetes Association Professional Practice Committee for Diabetes*, 2025[2]; Cusi et al., 2025[17]). Patients with a liver stiffness measurement (LSM) > 8.0 kPa or an ELF score > 9.8 should be referred to a hepatologist for further evaluation (Rinella et al., 2023[62]; EASL et al., 2024[19]; American Diabetes Association Professional Practice Committee for Diabetes*, 2025[2]; Cusi et al., 2025[17]). Similarly, individuals with FIB-4 scores > 2.67 are at high risk of advanced liver fibrosis and should be directly referred to a hepatologist for further assessment (Rinella et al., 2023[62]; EASL et al., 2024[19]; American Diabetes Association Professional Practice Committee for Diabetes*, 2025[2]; Cusi et al., 2025[17]).

However, in patients with T2DM, an FIB-4 score below 1.3 may be insufficient, as the FIB-4 index alone may miss advanced liver disease, especially in those with coexisting obesity and elevated serum ALT levels. In a study of 1,203 Italian outpatients with T2DM who underwent VCTE assessment, Mantovani et al. reported that the FIB-4 index had a sensitivity of 50.4 %, specificity of 66.3 %, negative predictive value (NPV) of 83.3 %, and positive predictive value (PPV) of 28.6 % for detecting significant liver fibrosis (defined as LSM ≥ 8 kPa) (Mantovani et al., 2026[41]). Although most T2DM patients with low FIB-4 score (< 1.3) had LSM < 8 kPa (83.3 %), 16.7 % still had significant liver fibrosis (LSM ≥ 8 kPa) (Mantovani et al., 2026[41]). In the intermediate (1.3-2.67) and high-risk (>2.67) FIB-4 groups, 25.7 % and 37.3 % of patients with T2DM, respectively, had LSM ≥ 8 kPa (Mantovani et al., 2026[41]). Similarly, in cohorts from Calgary (8,126 patients with MASLD; 34 % with T2DM) and Edmonton (985 MASLD patients; 22 % with T2DM), Shaheen et al. reported that patients with T2DM had a significantly higher probability of LSM above 8 kPa, even at FIB-4 scores below 1.3 or between 1.30 and 2.67, compared with those without diabetes (Shaheen et al., 2026[72]). Recently, Chen et al. evaluated this two-step approach for predicting advanced liver fibrosis (using a liver biopsy cohort of Chinese patients with T2DM and histologically confirmed MASLD) and for assessing the risk of long-term major adverse liver-related events in patients with T2DM (using the international VCTE-Prognosis cohort, including patients with T2DM and MASLD who underwent VCTE at 16 centers in the USA, Europe, and Asia) (Chen et al., 2026[13]). These investigators found that the noninvasive two-step approach, using the FIB-4 index followed by VCTE-assessed LSM, effectively stratified MASLD-related advanced fibrosis and the long-term risk of adverse liver-related events in individuals with T2DM. They also found that applying LSM cut-offs of 10 kPa and 15 kPa further optimized risk stratification for future adverse liver-related events (Chen et al., 2026[13]).

Collectively, these findings suggest that in individuals with T2DM, an FIB-4 score below 1.30 may be interpreted differently than in those without diabetes, underscoring the need to improve referral pathways when using the FIB-4 index as a first-line noninvasive test for people with T2DM.

## Screening for T2DM In Patients With MASLD

Based on the evidence discussed above, screening for T2DM in individuals with MASLD is clinically essential (Rinella et al., 2023[62]; EASL et al., 2024[19]). Early detection of dysglycemia enables timely intervention to prevent liver-related and extrahepatic complications. All individuals with MASLD should undergo systematic screening for T2DM at the time of diagnosis and periodically thereafter (Figure 1(Fig. 1), Panel B). Recommended laboratory tests include fasting plasma glucose, glycated hemoglobin (HbA1c), and, when appropriate, a standard oral glucose tolerance test, which may improve detection of early abnormalities in glucose metabolism (Rinella et al., 2023[62]; EASL et al., 2024[19]). Particular attention should be given to individuals with multiple cardiometabolic risk factors, such as overweight/obesity, hypertension, atherogenic dyslipidemia, or prediabetes. Given the progressive nature of MASLD and T2DM, regular reassessment of plasma glucose parameters is necessary. Early diagnosis and good glycemic control may also help slow the progression of liver disease and reduce overall CVD risk.

## Treatment

Effective management of MASLD in individuals with T2DM requires addressing not only hepatic steatosis, inflammation, and fibrosis but also the broader cardiometabolic risk profile that drives disease progression and long-term hepatic and extrahepatic complications (Chouik et al., 2025[15]; Zhou et al., 2026[93]). In this context, lifestyle interventions and emerging metabolism-based therapies are key pillars of MASLD management in individuals with and without T2DM.

### Lifestyle intervention

Lifestyle modification is the foundation of MASLD treatment for individuals living with T2DM (Rinella et al., 2023[62]; EASL et al., 2024[19]). Weight loss achieved through a weight-reducing diet and increased physical activity is the most effective non-pharmacological approach to improving liver-related outcomes (Rinella et al., 2023[62]; EASL et al., 2024[19]). Sustained weight loss of ≥ 5 % improves hepatic steatosis, whereas greater weight loss (7-10 % or > 10 %) is required to achieve histologic resolution of MASH and improve liver fibrosis (Rinella et al., 2023[62]; EASL et al., 2024[19]). Caloric restriction, especially with a Mediterranean-diet pattern or a hypocaloric diet, is recommended for its beneficial effects on insulin resistance, plasma lipid profile, and CVD risk (Rinella et al., 2023[62]; EASL et al., 2024[19]). Reducing intake of saturated fats, refined carbohydrates, and fructose is especially important for individuals with T2DM (Rinella et al., 2023[62]; EASL et al., 2024[19]). Regular physical activity, including aerobic and resistance training, further improves insulin resistance and reduces liver fat, even without significant weight loss (Rinella et al., 2023[62]; EASL et al., 2024[19]). In addition to weight management, lifestyle interventions should address broader cardiometabolic risk factors, including dysglycemia, dyslipidemia, and hypertension (Rinella et al., 2023[62]; EASL et al., 2024[19]). Avoiding alcohol and limiting dietary fructose intake are also recommended (Rinella et al., 2023[62]; EASL et al., 2024[19]). Behavioral support and structured programs may improve adherence and promote long-term sustainability. Overall, lifestyle interventions not only improve liver-related outcomes but also reduce the risk of CVD events, which remain the leading cause of morbidity and mortality in individuals with MASLD and T2DM (Targher et al., 2021[75], 2025[79]; Tilg et al., 2026[82]).

### Incretin-based therapy

GLP-1RAs, also known as incretin mimetics, have profoundly reshaped the management of T2DM worldwide (Targher et al., 2023[76], 2025[77]). These agents are well established as safe and effective for lowering blood glucose, and several compounds also demonstrate meaningful cardiovascular and renal benefits (Targher et al., 2023[76], 2025[77]). Recently, a new generation of incretin-based therapies has emerged, including dual agonists that target the GLP-1/GIP receptors (e.g., tirzepatide) or the GLP-1/glucagon receptors (e.g., cotadutide and survodutide), as well as triple agonists that act on the GLP-1, GIP, and glucagon receptors (e.g., retatrutide) (Targher et al., 2023[76], 2025[77]). These agents show great promise for improving glycemic control and reducing body weight.

Given the beneficial metabolic effects of GLP1RAs, randomized controlled trials (RCTs) in individuals with MASLD or MASH, regardless of T2DM status, have increasingly focused on assessing their hepatoprotective effects (Targher et al., 2023[76], 2025[77]). To date, subcutaneous semaglutide 2.4 mg/week is the first GLP-1RA approved by the US Food and Drug Administration (FDA) in August 2025 and by the European Medicines Agency (EMA) in March 2026 for the treatment of adults with non-cirrhotic MASH and moderate-to-advanced liver fibrosis (stage F2-F3). In the phase 3 placebo-controlled ESSENCE trial, 1,197 obese patients with biopsy-confirmed MASH and fibrosis stage F2-F3 (56 % with pre-existing T2DM) were randomized to receive once-weekly subcutaneous semaglutide 2.4 mg or placebo for 72 weeks (Sanyal et al., 2025[69]). Histological resolution of MASH without worsening of liver fibrosis occurred in 62.9 % of participants in the semaglutide group, compared with 34.3 % in the placebo group (P < 0.001) (Sanyal et al., 2025[69]). Additionally, significant improvement in liver fibrosis without worsening of MASH was observed in 36.8 % of those in the semaglutide group, compared with 22.4 % in the placebo group (P < 0.001) (Sanyal et al., 2025[69]). Subgroup analyses supported the efficacy of semaglutide 2.4 mg/week in improving MASH and liver fibrosis, regardless of T2DM status (Sanyal et al., 2025[69]).

Consistent with these findings, an updated meta-analysis of thirteen phase 2 and phase 3 RCTs (n = 1,811 participants) showed that GLP-1RAs, especially semaglutide 2.4 mg weekly, significantly reduce hepatic fat content, promote MASH resolution, and improve liver fibrosis in patients with MASH, regardless of T2DM status (Mantovani et al., 2025[42]). Additionally, a meta-analysis of 11 retrospective active-comparator, new-user cohort studies, using aggregate data from almost 1.5 million individuals with T2DM (647,903 GLP-1RA new users and 819,317 non-users), found that GLP-1RA use was associated with a significantly lower risk of major adverse liver-related outcomes and hepatic decompensation events (Celsa et al., 2025[10]). Consistent with this, the recent ADA guidelines strongly recommend GLP-1RA use, particularly semaglutide 2.4 mg/week, as the preferred therapeutic option for adults with T2DM and MASH, or for those at high risk of advanced liver fibrosis (American Diabetes Association Professional Practice Committee for Diabetes*, 2025[2]).

Phase 2b RCTs have also evaluated other incretin-based therapies for MASH across fibrosis stages (Targher et al., 2023[76], 2025[77]). Tirzepatide, a dual GIP and GLP-1 receptor agonist, has been shown to reduce hepatic steatosis in MASLD (Targher et al., 2023[76], 2025[77]). In a phase 2b, dose-finding, randomized, placebo-controlled trial (SYNERGY-NASH trial) involving 190 obese participants with biopsy-confirmed MASH and F2-F3 fibrosis, once-weekly subcutaneous tirzepatide (at weekly doses of 5, 10, or 15 mg) administered for 52 weeks resulted in MASH resolution without worsening of liver fibrosis in 44 %, 56 %, and 62 % of participants, respectively, compared with 10 % in the placebo group (P < 0.001 for all comparisons with placebo) (Loomba et al., 2024[38]). Improvement in at least one fibrosis stage without worsening of MASH occurred in 55 %, 51 %, and 51 % of participants in the three tirzepatide groups, compared with 30 % in the placebo group (Loomba et al., 2024[38]). These findings warrant further investigation in phase 3 clinical trials.

Survodutide, a dual GLP-1 and glucagon receptor agonist currently in development, has also shown promising results (Targher et al., 2023[76], 2025[77]). In addition, a 48-week phase 2b randomized, placebo-controlled trial (involving 293 adults with obesity and biopsy-confirmed MASH and fibrosis stage F1-F3) reported that once-weekly subcutaneous survodutide at doses of 2.4 mg, 4.8 mg, or 6.0 mg was superior to placebo for histological improvement in MASH without worsening of liver fibrosis, warranting further investigation in phase 3 trials (Sanyal et al., 2024[67]).

### Pioglitazone

Pioglitazone, a peroxisome proliferator-activated receptor-γ (PPAR-γ) agonist, has been extensively studied as a treatment for MASH, particularly in individuals with T2DM (Francque et al., 2021[24]; Mantovani et al., 2022[39]). By enhancing insulin sensitivity in adipose tissue, liver, and skeletal muscle, pioglitazone reduces hepatic lipotoxicity, inflammation, and downstream fibrogenic signaling, key drivers of MASH (Francque et al., 2021[24]; Mantovani et al., 2022[39]). Phase 2 RCTs have consistently shown that pioglitazone improves histological features of MASH (Mantovani et al., 2022[39]). In the landmark phase 3 placebo-controlled PIVENS trial and subsequent studies involving patients with and without T2DM, pioglitazone achieved MASH resolution in a significant proportion of treated individuals (Sanyal et al., 2010[68]). A meta-analysis of five phase 2b placebo-controlled trials found that pioglitazone treatment (up to 45 mg/day) for 6-24 months led to MASH resolution and may improve advanced liver fibrosis (Musso et al., 2017[53]). Some guidelines recommend pioglitazone for selected patients with T2DM and biopsy-proven MASH (Rinella et al., 2023[62]; American Diabetes Association Professional Practice Committee for Diabetes*, 2025[2]). Furthermore, combination therapy with pioglitazone *plus* a GLP-1RA has been reported to be safe and effective for treating chronic hyperglycemia, reducing overall mortality and CVD events, and improving MASLD and fibrosis in individuals with T2DM (American Diabetes Association Professional Practice Committee for Diabetes*, 2025[2]; Florez et al., 2025[23]). However, despite its efficacy, pioglitazone raises some concerns about moderate weight gain (especially in subcutaneous rather than visceral fat), fluid retention, and potential long-term safety issues, warranting careful patient selection and monitoring (American Diabetes Association Professional Practice Committee for Diabetes*, 2025[2]).

### SGLT2 inhibitors and other glucose-lowering agents

SGLT2 inhibitors have been shown to reduce hepatic steatosis (Mantovani et al., 2021[47]), likely by promoting weight loss, improving insulin resistance, and modulating energy balance (Scheen, 2019[70]; Mantovani et al., 2022[39]). However, their effects on MASH remain uncertain, as robust clinical trial evidence with histological liver endpoints is lacking (Mantovani et al., 2021[47], 2022[39]). Recently, in a small phase 3 randomized, placebo-controlled trial of 154 Chinese adults with biopsy-confirmed MASH, with or without T2DM, dapagliflozin 10 mg daily for 48 weeks was more effective than placebo for MASH resolution (23 % of those assigned to dapagliflozin vs. 8 % of those assigned to placebo) and for improvement in liver fibrosis (45 % vs. 20 %) (Lin et al., 2025[36]). A recent meta-analysis of eight active-comparator, new-user cohort studies, including 626,104 patients with T2DM (397,806 new users of SGLT2 inhibitors and 228,298 new users of other glucose-lowering agents), reported that SGLT2 inhibitor use was associated with a significantly lower risk of major adverse liver-related outcomes and liver-related deaths over a median follow-up of 2.7 years (Mantovani et al., 2025[43]). In individuals with T2DM and MASLD, glucose-lowering medications other than GLP-1RAs or pioglitazone, such as sulfonylureas, dipeptidyl peptidase-4 inhibitors, acarbose, or insulin, can be used for glycemic management when clinically indicated (American Diabetes Association Professional Practice Committee for Diabetes*, 2025[2]). However, because these glucose-lowering medications have not been evaluated in randomized clinical trials with adequate liver histological endpoints, their long-term effects on MASH and liver fibrosis remain undefined (Mantovani et al., 2022[39]).

### Resmetirom

Resmetirom is an orally administered, liver-directed, selective thyroid hormone receptor-β (THR-β) agonist effective for treating MASH and liver fibrosis (Petta et al., 2024[60]). By selectively targeting THR-β in hepatocytes, resmetirom enhances hepatic lipid metabolism while minimizing off-target effects typically associated with thyroid hormone activity, such as cardiovascular and bone toxicity mediated by THR-α activation (Petta et al., 2024[60]). Resmetirom's mechanisms of action include increased fatty acid β-oxidation, reduced hepatic *de novo* lipogenesis, and enhanced clearance of plasma atherogenic lipoproteins, thereby targeting key pathways involved in MASH development (Petta et al., 2024[60]).

The pivotal phase 3 placebo-controlled MAESTRO-NASH trial evaluated the efficacy and safety of resmetirom in 966 obese individuals with biopsy-confirmed MASH and fibrosis stages F2-F3 (Harrison et al., 2024[29]). In this trial, both the 80-mg and 100-mg doses of resmetirom for 52 weeks were superior to placebo for MASH resolution (25.9 % of patients in the 80-mg resmetirom group vs. 29.9 % in the 100-mg resmetirom group vs. 9.7 % in the placebo group; P < 0.001 for both comparisons with placebo) and for improvement in liver fibrosis by at least one stage (24.2 % of those in the 80-mg resmetirom group vs. 25.9 % of those in the 100-mg resmetirom group vs. 14.2 % of those in the placebo group; P < 0.001) (Harrison et al., 2024[29]). Subgroup analyses supported the efficacy of resmetirom in improving MASH and liver fibrosis, regardless of T2DM status (Harrison et al., 2024[29]). Other analyses confirmed the robustness of these results (Ciardullo and Mantovani, 2024[16]). In addition to benefits in liver histology, resmetirom also had favorable effects on plasma lipid profile, with significant reductions in atherogenic lipids, including plasma LDL cholesterol, apolipoprotein B, triglycerides, and lipoprotein(a) levels (Harrison et al., 2024[29]; Petta et al., 2024[60]). These reductions are particularly relevant given the increased risk of CVD in patients with MASLD. From a safety perspective, resmetirom is generally well tolerated (Harrison et al., 2024[29]). The most commonly reported adverse events are transient, mild-to-moderate gastrointestinal symptoms, such as diarrhea and nausea, particularly during the initial treatment phase (Harrison et al., 2024[29]).

Importantly, no significant safety signals related to thyroid axis disruption, cardiac arrhythmias, or bone metabolism have been identified to date, supporting resmetirom's liver-selective profile (Harrison et al., 2024[29]). Notably, based on findings from the phase 3 MAESTRO-NASH trial, resmetirom received conditional approval from the FDA (March 2024) and the EMA (August 2025) for the treatment of adults with noncirrhotic MASH and moderate-to-advanced fibrosis, marking a significant milestone in the field. Candidates for resmetirom treatment are adults with MASH and liver fibrosis but without cirrhosis or other active liver conditions, regardless of T2DM status (Petta et al., 2024[60]; Chen et al., 2025[12]). Despite these advances, several questions remain, including the long-term durability of the histological response, effects in patients with cirrhosis, and the potential benefits of combination therapy with other agents, such as semaglutide and other incretin-based therapies (Noureddin et al., 2025[56]; Polyzos et al., 2026[61]; Zhou et al., 2026[92]). Ongoing and future trials will help clarify the optimal positioning of resmetirom within the therapeutic landscape of MASH.

**Figure 2:**
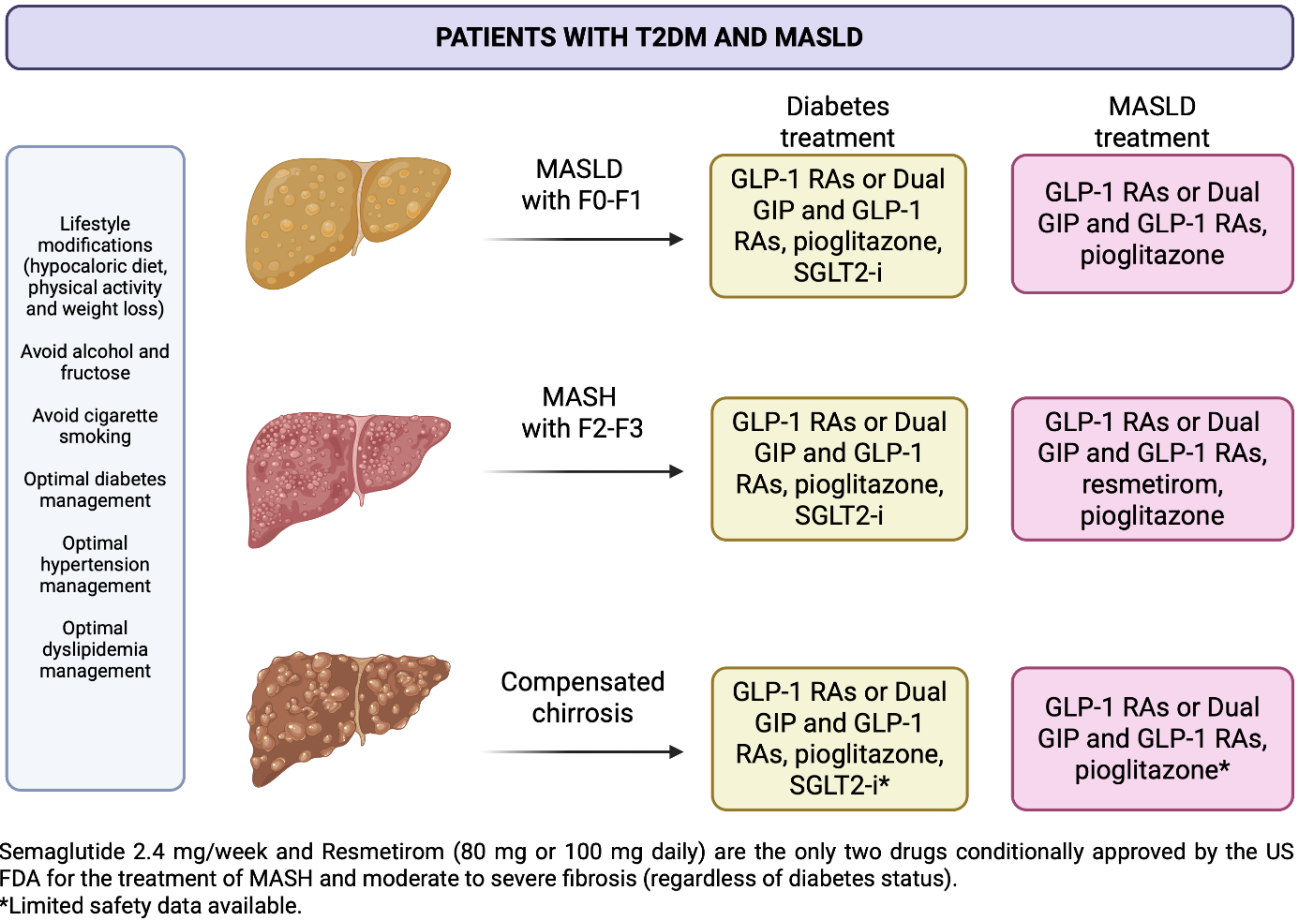
Proposed treatment algorithm for individuals with type 2 diabetes (T2DM) and MASLD, based on current scientific guidelines. Created with https://BioRender.com. Abbreviations: F0-F1, no to minimal fibrosis; F2-F3, moderate fibrosis; GIP, glucose-dependent insulinotropic polypeptide; GLP-1RA, glucagon-like peptide 1 receptor agonist; MASH, metabolic dysfunction-associated steatohepatitis; SGLT2i, sodium-glucose cotransporter 2 inhibitor

Figure 2(Fig. 2) shows a possible treatment algorithm for individuals with T2DM and MASLD or MASH based on current guidelines (Rinella et al., 2023[62]; EASL et al., 2024[19]; American Diabetes Association Professional Practice Committee for Diabetes*, 2025[2]). For patients with T2DM and MASLD or MASH, treatment should preferably include GLP-1-based therapies alone or in combination with SGLT2 inhibitors, which have been shown to improve MASLD and MASH, in addition to their established effects on cardiorenal risk reduction. Other molecules, such as lanifibranor (a pan-PPAR agonist) (Francque et al., 2021[25]) and fibroblast growth factor 21 (FGF-21) analogues (Mantovani et al., 2024[50]; Noureddin et al., 2025[57]), have been investigated in phase 2 RCTs for treating MASH and liver fibrosis. However, although the available data are promising, they remain preliminary and insufficiently robust to support inclusion in a treatment algorithm, particularly in patients with T2DM.

## Conclusions

The relationship between MASLD and T2DM is complex, bidirectional, and clinically significant, reflecting a shared metabolic milieu characterized by adipose tissue dysfunction, insulin resistance, low-grade inflammation, dysregulated lipid metabolism, and intestinal dysbiosis. Rather than viewing them as separate conditions, MASLD and T2DM should be understood as interconnected components of a broader multisystem metabolic disorder, each capable of triggering and exacerbating the other.

**Figure 3:**
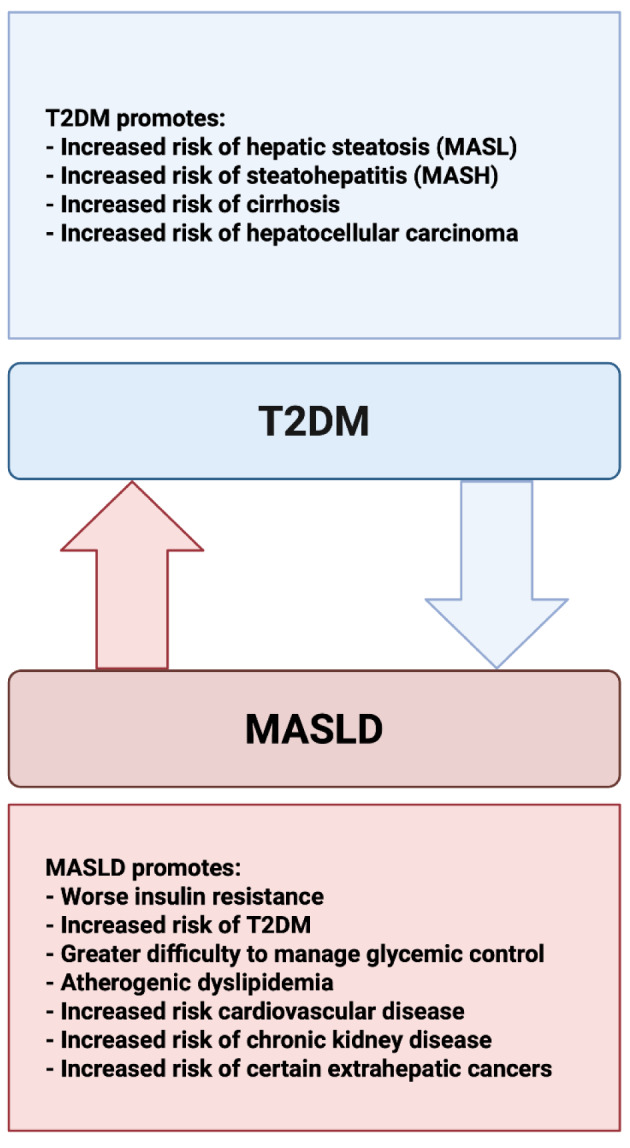
The “vicious circle” linking type 2 diabetes mellitus (T2DM) and MASLD

Strong evidence shows that T2DM significantly increases the prevalence and severity of MASLD, accelerating progression to advanced fibrosis, cirrhosis, liver failure, and HCC. At the same time, MASLD increases the risk of incident T2DM, particularly in individuals with more severe liver fibrosis. The close interrelationship between MASLD and T2DM creates a self-perpetuating vicious cycle that amplifies the risk of both hepatic and extrahepatic complications, including fatal and nonfatal CVD events and chronic kidney disease, which are major causes of morbidity and mortality in this patient population (as summarized in Figure 3(Fig. 3)).

From a clinical perspective, these findings underscore the importance of an integrated, proactive approach to screening and managing MASLD and T2DM (Mantovani and Valenti 2021[51]; Targher et al. 2021[78]). Systematic assessment of liver fibrosis in individuals with T2DM and routine evaluation of glucose metabolism in those with MASLD are crucial for early detection and risk stratification. Although current noninvasive tools for liver fibrosis are helpful, they require refinement to improve diagnostic accuracy in high-risk populations, including those with T2DM.

Lifestyle modification remains the cornerstone of treatment, with weight loss and physical activity providing significant benefits for both liver disease and glycemic control. To date, subcutaneous semaglutide 2.4 mg/week and resmetirom (80 mg or 100 mg orally once daily) are the only two medications conditionally approved by the FDA and EMA for the treatment of adults with non-cirrhotic MASH and moderate-to-advanced liver fibrosis, regardless of the presence of T2DM.

Future research should focus on clarifying the mechanisms underlying the close bidirectional relationship between MASLD and T2DM, improving risk prediction models, and developing combination therapies tailored to individual patients. Ultimately, a multidisciplinary, patient-centered, and holistic approach is crucial to effectively addressing the increasing global burden of MASLD and T2DM and to reducing the long-term risk of hepatic and extrahepatic clinical outcomes.

## Declaration

### Funding

GT was supported in part by grants from the School of Medicine, University of Verona, Verona, Italy.

### Conflict of interest

Nothing to declare.

### Artificial Intelligence (AI) - assisted technology

We did not use any AI tools.
